# Heritability of Crohn’s disease and ulcerative colitis: a Swedish nationwide population-based twin study

**DOI:** 10.1093/ecco-jcc/jjag044

**Published:** 2026-04-22

**Authors:** Olle Grännö, Joel Thunberg, Jonas F Ludvigsson, Ralf Kuja-Halkola, Carl Mårten Lindqvist, Jonas Halfvarson

**Affiliations:** Department of Laboratory Medicine, Clinical Microbiology, Faculty of Medicine and Health, Örebro University, Örebro, Sweden; Department of Gastroenterology, Faculty of Medicine and Health, Örebro University, Örebro, Sweden; Department of Medical Epidemiology and Biostatistics, Karolinska Institutet, Solna, Sweden; Department of Pediatrics, Örebro University Hospital, Örebro, Sweden; Department of Medicine, Columbia University College of Physicians and Surgeons, New York, NY, United States; Department of Medical Epidemiology and Biostatistics, Karolinska Institutet, Solna, Sweden; Faculty of Medicine and Health, School of Medical Sciences, Örebro University, Örebro, Sweden; Department of Gastroenterology, Faculty of Medicine and Health, Örebro University, Örebro, Sweden

**Keywords:** inflammatory bowel disease, heritability, twin study

## Abstract

**Background and Aims:**

Limited statistical power has hampered previous estimates of concordance between relatives and heritability in inflammatory bowel diseases (IBD). To examine the genetic component of Crohn’s disease and ulcerative colitis, we established the largest nationwide IBD twin cohort to date and assessed estimates of concordance and heritability.

**Methods:**

We used the Swedish Twin Registry to identify all twins from complete pairs with known zygosity born between 1886 and 2004. The Swedish National Patient Register was used to identify all patients diagnosed with IBD. We calculated proband concordance rates and fitted a model estimating explained variance in diseases due to genetics (ie, the heritability), environment shared between twins, and environment unique to each twin.

**Results:**

A cohort of 111 080 twins was followed until a median age of 62.2 years, during which 964 individuals were diagnosed with IBD. The proband concordance rate for Crohn’s disease was 0.30 in monozygotic pairs and 0.02 in dizygotic pairs. The corresponding rates for ulcerative colitis were 0.15 and 0.03. After adjusting for sex and birth year, heritability was estimated to be 0.78 (95% CI: 0.68-0.87) for Crohn’s disease and 0.57 (95% CI: 0.46-0.69) for ulcerative colitis.

**Conclusion:**

In this large population-based twin study, the heritability of Crohn’s disease was 0.78 and 0.57 for ulcerative colitis. These findings highlight the disparity between heritability estimates from twin studies and those inferred from genome-wide association studies, underscoring the need for continued exploration of the genetic basis of IBD.

## 1. Introduction

Inflammatory bowel disease (IBD), encompassing Crohn’s disease and ulcerative colitis, is a complex disease that results in abdominal pain, diarrhea and rectal bleeding. A prevailing theory of the etiology of IBD is that exposure to genetic and environmental risk factors translates into a dysbiotic gut microbiota, which triggers an aberrant immune response and chronic inflammation in the gastrointestinal tract.^1^^,^^2^

The first nationwide twin study demonstrated a strong genetic contribution in Crohn’s disease.^3^ In a cohort of 80 Swedish twin pairs, concordance rates were higher in monozygotic (MZ) pairs than in dizygotic (DZ) pairs.^3^ Since this study, several twin studies from Sweden, Great Britain, Denmark, Germany and Norway have sought to disentangle the relative contribution of genetic and environmental factors.^3–10^ Despite a prevalence of IBD ranging from 673 to 951 cases per 100 000 in these countries,^11^ only limited numbers of IBD-affected twins have been included in these cohorts.^3^^,^^5–7^^,^^12^ As a result, reported estimates of concordance rates are inconsistent, and confidence intervals (CIs) have rarely been presented (Supplementary Table S1—see online supplementary material).^3^^,^^5–7^^,^^12^ In addition to the limited statistical power of earlier studies, methodological limitations further challenge the interpretation of results. The duration of follow-up periods has been short, and additional IBD diagnoses would likely have been observed with longer follow-up. This truncation may have had a pronounced effect on observed concordance rates, as demonstrated in the follow-up of the first Swedish study.^7^ Many studies have reported rates of concordant pairs without considering the risk of ascertainment bias.^13^ Only two studies have examined heritability estimates for Crohn’s disease and ulcerative colitis separately.^3^^,^^7^

Large genome-wide association studies (GWAS) have identified over 240 genetic loci with moderate-to-subtle effects in line with the observed heritable effect in twin studies.^14^^,^^15^ However, identified risk loci explain only 26% and 17%-19% of variance in disease liability in Crohn’s disease and ulcerative colitis, respectively.^16^^,^^17^ The large gap between the inferred heritability from GWAS and the heritability estimates from twin studies suggests the importance of other mechanisms in the etiology of IBD (which are not captured in traditional GWAS), such as rare variants, epigenetic changes and the interplay between genetic variants and environmental exposure.^18^ Alternatively, previously reported heritability estimates from twin studies may have overestimated the influence of genetic predisposition due to imprecise measurements.

To examine the genetic component of Crohn’s disease and ulcerative colitis, we established the largest nationwide IBD twin cohort to date and assessed estimates of concordance and heritability.

## 2. Methods

### Study population

Using the Swedish Twin Registry (STR), we identified all twins from complete pairs with established zygosity, born up to 2004. Details about the STR is provided in the Supplementary Methods (see online supplementary material). For participants born before 1975, both twins within a pair were required to be alive by 31 December 1974 to ensure adequate representation of national health registries and to minimize misclassification of IBD status. Both same-sex and opposite-sex dizygotic pairs were included to maximize statistical power.

### Outcome assessment

Twins were followed until a diagnosis of IBD, death or end of the follow-up (31 March 2020), whichever occurred first. The Swedish National Patient Register (NPR) was used to identify all twins diagnosed with IBD. The NPR is known for its high validity and encompasses prospectively recorded individual-level data on hospital discharge diagnoses since 1964, with national coverage since 1987.^19^ Since 2001, information on non-primary outpatient visits has also been recorded.^19^ All diagnoses are coded according to the International Classification of Diseases and Related Health Problems (ICD). Crohn’s disease or ulcerative colitis was defined as having ≥ 2 IBD-associated ICD codes, corresponding to a positive predictive value of 93% (Supplementary Figure S1 and Supplementary  Tables S2-S5—see online supplementary material).^20^ In patients with mixed Crohn’s disease and ulcerative colitis codes, ICD codes and surgical procedure codes within the past 5 years of visits were used to assign the IBD subtype.^21^

### Statistics

Categorical variables were summarized as frequencies and proportions, and continuous variables as medians with interquartile range (IQR). Prevalence of IBD overall and by subtype was defined as the cumulative prevalence on March 31, 2020, calculated as the number of affected twins divided by the total number in the cohort. Age at diagnosis was defined by the date of the first IBD-associated diagnostic listing.

#### Concordance rates and tetrachoric correlations

Proband concordance rates were calculated separately for Crohn’s disease and ulcerative colitis using a generalized estimating equation with a log-link function and a cluster-robust sandwich estimator to account for within-pair dependencies. Further details are provided in the Supplementary Methods (see online supplementary material). Tetrachoric correlations were used to quantify the similarity in liability to Crohn’s disease and ulcerative colitis between MZ and DZ pairs, with higher correlations in MZ than DZ pairs indicating a genetic influence.

#### Heritability

Heritability estimates were derived using classical twin modeling. We applied the ACE model, partitioning variance into additive genetic (A), shared environmental (C), and non-shared environmental (E) effects. Additionally, we fitted alternative models, including CE, ADE and AE, with D representing dominant genetic effects. Full model-fitting details are provided in the Supplementary Methods (see online supplementary material). Analyses were repeated adjusting for sex and birth year, with birth year modeled as a quartic polynomial function separately for males and females.

#### Software

All statistical analyses were conducted in R (version 4.1.2, R core team, 2021) for Windows.^22^ Heritability was calculated using the “mets” package (version 1.2.9).^23^ We also used the “polycor” package (version 0.8-1)^24^ and the “drgee” package (version 1.1.10).^25^  *P*-values < .05 were considered statistically significant.

#### Ethical considerations

This study was approved by the Swedish Ethical Review Authority (registration number 2019-04802).

## 3. Results

**Figure 1.**
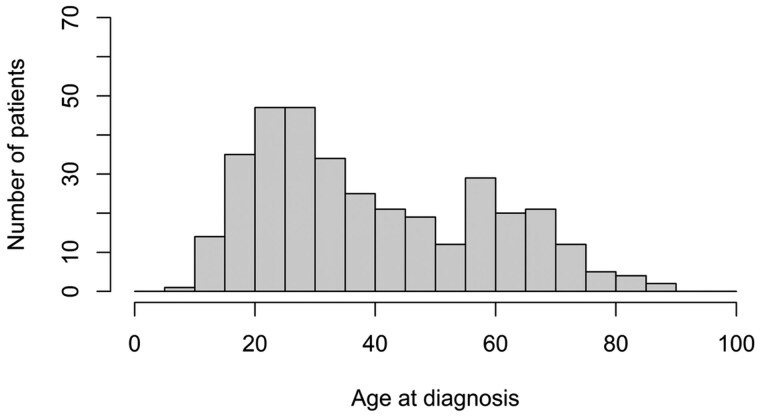
Distribution by age at diagnosis of Crohn’s disease.

**Figure 2.**
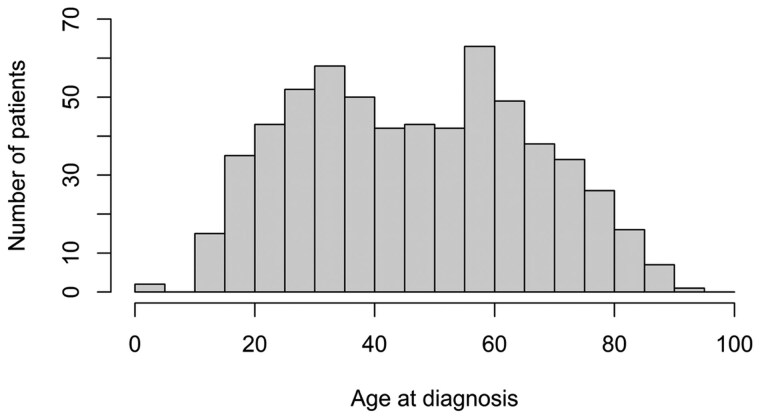
Distribution by age at diagnosis of ulcerative colitis.

A total of 111 080 twins were followed until a median age of 62.2 (IQR 29.2-77.2) years. 964 individual twins were diagnosed with IBD, resulting in a prevalence of 0.87%. Prevalence estimates for Crohn’s disease (*n* = 348) and ulcerative colitis (*n* = 616), stratified for sex and zygosity, are shown in Table 1 and Supplementary Table S6 (see online supplementary material). The median age at diagnosis was 33.7 (IQR 23.8-56.4) years for Crohn’s disease and 46.3 (IQR 30.5-61.8) years for ulcerative colitis. The distribution by age at diagnosis of each subtype of IBD is shown in Figures 1 and 2.

**Table 1.** Prevalence estimates for Crohn’s disease and ulcerative colitis in the Swedish nationwide twin cohort.

	Total number of twins, *n*	Twins diagnosed with CD, *n*	Prevalence of CD, %	Twins diagnosed with UC, *n*	Prevalence of UC, %	Median age at diagnosis, years (IQR)		Median age at the end of follow-up, years (IQR)	
						CD patients	UC patients	CD twin pairs	UC twin pairs
**All**	111 080	348	0.31	616	0.55	33.7 (23.8-56.4)	46.3 (30.5-61.8)	64.2 (42.2-75.2)	67.2 (51.2-77.2)
** Males**	52 176	166	0.32	306	0.59	35.9 (24.5-56.8)	46.3 (30.4-62.7)	64.2 (42.1-74.9)	67.2 (52.2-77.3)
** Females**	58 904	182	0.31	310	0.53	32.4 (22.9-56.2)	45.9 (30.5-61.3)	63.8 (42.2-75.2)	66.2 (52.2-76.2)
** Monozygotic**	36 090	100	0.28	178	0.49	35.3 (23.5-54.3)	36.2 (26.0-59.1)	66.2 (43.2-74.2)	61.2 (41.4-76.2)
** Dizygotic**	74 990	274	0.37	446	0.59	33.6 (24.1-56.8)	49.1 (33.0-62.7)	64.2 (42.1-75.2)	68.1 (57.1-77.2)

Abbreviations: CD: Crohn’s disease, UC: ulcerative colitis, IQR: interquartile range.

### Concordance rates and tetrachoric correlations

In Crohn’s disease and ulcerative colitis, a higher concordance rate was observed in MZ twin pairs than in DZ pairs (Table 2). The proband concordance rate for Crohn’s disease was 0.30 in MZ pairs and 0.02 in DZ pairs. The corresponding rates for ulcerative colitis were 0.15 and 0.03. In line with the observed proband concordance rates, higher tetrachoric correlations were observed for MZ pairs compared to DZ pairs (Table 2). These findings substantiate the role of genetic factors in the etiology of Crohn’s disease and ulcerative colitis.

**Table 2.** Concordance rates and tetrachoric correlations for Crohn’s disease and ulcerative colitis in a Swedish nationwide twin cohort.

	Unaffected pairs	Discordant pairs	Concordant pairs	Proband concordance rate (95% CI)	Tetrachoric correlations (95% CI)
**Monozygotic—Crohn’s disease**	17 960	70	15	0.30 (0.20-0.44)	0.79 (0.71-0.88)
**Dizygotic—Crohn’s disease**	37 249	244	2	0.02 (0.0-0.06)	0.20 (0.0-0.41)
**Monozygotic—ulcerative colitis**	17 880	152	13	0.15 (0.09-0.24)	0.59 (0.48-0.70)
**Dizygotic—ulcerative colitis**	37 064	424	7	0.03 (0.02-0.07)	0.25 (0.13-0.38)

### Heritability

The heritability estimate for Crohn’s disease, adjusted for sex and birth year, was 0.78 (95% CI: 0.68-0.87). Non-shared environmental effects (E) explained the remaining variation in liability, *E* = 0.22 (95% CI: 0.13-0.32). Compared to Crohn’s disease, we observed a lower adjusted heritability estimate for ulcerative colitis [0.57 (95% CI: 0.46-0.69); *P*-value for difference = .02], while non-shared environmental effects (E) were estimated to be 0.43 (95% CI: 0.31-0.54). Only minor differences were observed between the crude and adjusted estimates for Crohn’s disease and ulcerative colitis (Supplementary Tables S7 and S8—see online supplementary material). The ADE and AE models yielded heritability estimates comparable to those obtained from the ACE model.

## 4. Discussion

In this nationwide twin study of 111 080 participants, we confirmed that Crohn’s disease is associated with a greater genetic risk than ulcerative colitis. Among 927 twin pairs affected with IBD, proband concordance rates were markedly higher in MZ pairs compared with DZ pairs. The observed rates translated into heritability estimates of 0.78 (95% CI: 0.68-0.87) for Crohn’s disease and 0.57 (95% CI: 0.46-0.69) for ulcerative colitis. While earlier studies suggested a greater genetic influence in Crohn’s disease than ulcerative colitis, this is the first study to demonstrate a statistically significant difference between the two subtypes of IBD. By leveraging a substantially larger twin dataset than previously available and applying contemporary twin modeling techniques, we were able to provide precise heritability estimates with confidence intervals, supporting the generalizability of our findings. These results are directly relevant to ongoing molecular genetic studies, as they provide a benchmark for quantifying the proportion of heritability explained by known genetic variants and highlight the extent of the remaining, so-called “missing heritability”.

Several twin studies have reported concordance estimates for IBD.^3–10^^,^^12^ However, differences in study design and reported estimates of genetic predisposition challenge the interpretation and comparison of these estimates. Most studies have only reported pairwise concordance rates, representing the proportion of pairs in which both twins are affected (concordant for the disease). Comparisons of pairwise concordance rates across studies are inappropriate because the measure is susceptible to ascertainment bias.^13^

Unlike pair concordance, proband concordance reflects the risk of disease in the co-twin of an affected twin (the proband) and is therefore more suitable for cross-study comparisons. Only five population-based twin studies, all linking data from the Swedish or the Danish twin registry with national patient registers, have reported proband concordance rates.^3^^,^^5–7^^,^^12^ However, the observed rates vary even within these studies.

In the most recent follow-up of these cohorts, higher proband concordance rates have been reported for MZ Crohn’s disease and DZ ulcerative colitis compared to the findings presented in this study. Jess et al. reported a proband concordance rate for Crohn’s disease of 0.64 in MZ pairs and a rate of 0.05 for ulcerative colitis in DZ pairs among 103 Danish IBD twin pairs born between 1964 and 1982.^6^ The corresponding rates were 0.38 and 0.08 based on the most recent follow-up of 173 Swedish pairs born from 1886 to 1980.^7^ By comparison, we observed a proband concordance rate of 0.30 for Crohn’s disease in MZ pairs and 0.03 for ulcerative colitis in DZ pairs. Differences in follow-up duration, attained age, IBD definitions, study periods, and applied statistical models may account for these discrepancies.

Given the wide age range of IBD diagnoses, sufficient follow-up duration is critical for accurately estimating concordance rates from twin studies. In our study, twins were followed until a median age of 62.2 years, whereas previous Swedish and Danish twin cohorts attained substantially younger ages at follow-up.^6^^,^^7^ A sufficient follow-up time may be critical to obtain robust concordance estimates, as a substantial lag time can exist between IBD diagnoses, even in monozygotic, IBD-concordant twin pairs. Differences in age at IBD diagnosis within monozygotic twin pairs likely reflect varying rates of risk accumulation, potentially driven by differential exposure to environmental risk factors. Earlier studies also relied on retrospective review of medical notes,^6^^,^^7^ which may have been constrained by incomplete or missing historical information. In contrast, we applied register-based definitions for IBD, capitalizing on the Swedish NPR well-established accuracy and high positive predictive value for IBD.^19^^,^^20^^,^^26^ The advent of advanced diagnostic tools, such as high-resolution endoscopes, magnetic resonance imaging and capsule endoscopies, has also enhanced the ability to diagnose IBD in recent decades.

We also estimated the heritability for Crohn’s disease and ulcerative colitis. Heritability reflects the relative contribution of genetic inheritance to the observed variation in a population trait. Heritability estimates, ranging from 0 to 1, indicate the degree to which a trait is influenced by genetic factors within a population, with values closer to 1 signifying a higher degree of heritability. A higher estimate was observed for Crohn’s disease (0.78) than for ulcerative colitis (0.57). Despite the importance of heritability as a metric, estimates of heritability for Crohn’s disease and ulcerative colitis have only been reported in two studies of Swedish twins^3^^,^^7^ and one meta-analysis.^16^ The most recent Swedish study was published in 2011 and reported a heritability estimate of 0.89 for Crohn’s disease and 0.23 for ulcerative colitis.^7^ In the current study, the difference in heritability between Crohn’s disease and ulcerative colitis was smaller, which is consistent with molecular studies demonstrating that a substantial proportion of genetic risk loci are shared between the two IBD subtypes.^27^^,^^28^ In line with these results, previous studies have reported sibling and twin pairs with mixed IBD subtypes, in which one individual develops Crohn’s disease and the other ulcerative colitis.^7^^,^^29^ Family studies suggest that environmental exposures such as smoking can interact with genetic susceptibility to influence IBD subtype. Bridger et al. observed that in 21 of 23 sibling pairs discordant for IBD subtype and smoking patterns, Crohn’s disease occurred in the smoker and ulcerative colitis in the non-smoker.^29^ Shifts in IBD subtypes have also been observed across generations in families with a high genetic risk of IBD, further supporting a shared genetic predisposition.^30^ Although the mechanisms underlying IBD subtype divergence are not fully elucidated, differences in exposure to environmental risk factors such as smoking offer a plausible explanation.^29^ In the meta-analysis of pooled data from all published twin studies on IBD until 2014, the heritability estimates were 0.75 for Crohn’s disease and 0.67 for ulcerative colitis.^16^ The estimates derived from the pooled analysis may be subject to bias, as a large proportion of the twins included in the meta-analysis were identified through questionnaires specifically targeting individuals with IBD. Because twins concordant for a disease may be more prone to answer such questionnaires, this practice may result in biased estimates. The heritability estimates observed in this study (0.78 for Crohn’s disease and 0.57 for ulcerative colitis) differed from previous estimates. In contrast to the meta-analysis, identifying twins and classifying IBD status was based on independently gathered data from national registers, reducing the risk of introducing selection bias. In addition, we employed structural equation modeling to assess heritability, as it permits adjustments for covariates such as age and birth year, while previous studies have used different methods.^3^^,^^7^^,^^16^ Nevertheless, heritability and concordance rates are population-specific estimates that may vary over time and across geographic regions because of differences in environmental exposures.

Accurate estimations of heritability are critical for research on molecular genetics.^31^ In GWAS, the total risk increase attributed to all single nucleotide polymorphisms (SNPs) can be used to estimate heritability without information about kinship. However, SNP heritability for Crohn’s disease and ulcerative colitis only explains a fraction of the heritability estimated from most family and twin studies.^16^ This discrepancy, known as the “missing heritability paradox”, persists despite large GWAS consortia and advances in statistical methods.^18^ Several explanations have been proposed, including the contribution of rare genetic variants, epigenetic regulation, gene-gene interactions (epistasis) and gene-environment interactions.^32^ Direct comparisons of heritability estimates between GWAS and family studies have been inconclusive because of the limited sample size of previous twin studies. Our study is the first to present heritability estimates for Crohn’s disease and ulcerative colitis with robust confidence intervals, enabling evaluation of statistical uncertainty. The lower bounds of the 95% CIs were 0.68 for Crohn’s disease and 0.46 for ulcerative colitis. These figures can be compared to the point estimates of SNP heritability at 0.20 and 0.13 reported in a recent GWAS including ∼30 000 IBD cases.^14^ Even with this conservative approach, a substantial gap persists between the heritability estimates from the present study and the GWAS estimates. This observation has significant implications for GWAS, whole-genome or exome sequencing initiatives, as it suggests that a substantial portion of the genetic basis underlying IBD remains to be explored.

### Strength and limitations

The main strength of this study is the large sample size, permitting more precise estimates of heritability. With a sample size of 927 twin pairs discordant or concordant for IBD, this study has superior statistical power compared to the previously largest cohort of 173 IBD twin pairs.^7^ An additional strength of our study lies in its adjustment for birth year, as variations in follow-up duration and diagnostic practices across decades may impact the likelihood of an IBD diagnosis. The use of ≥ 2 IBD-associated ICD codes in the NPR provided a case definition with a positive predictive value of 93%, further reinforcing the robustness of our IBD definition.

Nonetheless, a more extended follow-up period may be necessary for twin pairs born in recent decades. To account for this, we adjusted for birth year when computing heritability, which only minimal affected the estimates. While some misclassification between Crohn’s disease and ulcerative colitis may have occurred and some IBD diagnoses may have been missed due to the lack of outpatient records in the NPR before 2004, such misclassification is likely non-differential between MZ and DZ twins, limiting its influence on heritability estimates. Despite the large sample size of 55 540 twin pairs, confidence intervals for some estimates remained comparatively wide, underscoring the necessity of future meta-analyses integrating data from multiple unselected twin cohorts. A limitation of this study is the lack of data on molecular genetics and perinatal risk factors that may shape disease risk within twin pairs, including detailed information on birth order and chorionicity. Future studies integrating molecular genetic and perinatal data will be required to enable a more detailed characterization of IBD risk in twins.

## 5. Conclusions

This large-scale population-based twin study found heritability estimates for Crohn’s disease and ulcerative colitis of 0.78 and 0.57, respectively. These findings confirm a greater genetic contribution to Crohn’s disease than to ulcerative colitis and demonstrate that a significant discrepancy persists between the heritability estimated in twin studies and the inferred heritability from GWAS (ie, missing heritability). Importantly, this gap implies that a significant proportion of the genetic landscape of IBD remains to be discovered. Larger GWAS combined with advances in sequencing techniques and analytic methods may be required to further elucidate the missing heritability of IBD.

## Supplementary Material

jjag044_Supplementary_Data

## Data Availability

Under current data protection legislation, the data sets used for this paper cannot be shared directly. They must be requested from the respective registry holders after approval by the Swedish Ethical Review Authority.
