# Supplementary material for: Heritability of Crohn’s disease and ulcerative colitis: a Swedish nationwide population-based twin study
Source: J Crohns Colitis. 2026 Apr 22;20(4):jjag044. doi: 10.1093/ecco-jcc/jjag044 (PMC13102174; doi:10.1093/ecco-jcc/jjag044)
Supplement: jjag044_Supplementary_Data [file jjag044_supplementary_data.docx]

**Supporting Information**

**Heritability of Crohn’s disease and ulcerative colitis: A Swedish nationwide population-based twin study**

Olle Grännö^1^, Joel Thunberg^2^, Jonas F Ludvigsson^3-5^, Ralf Kuja-Halkola^3^, Carl Mårten Lindqvist*^6^, Jonas Halfvarson*^2^

*Should be considered joint senior author

**Affiliations**:

1. Department of Laboratory Medicine, Clinical Microbiology, Faculty of Medicine and Health, Örebro University, Örebro, Sweden

2. Department of Gastroenterology, Faculty of Medicine and Health, Örebro University, Örebro, Sweden

3. Department of Medical Epidemiology and Biostatistics, Karolinska Institutet, Solna, Sweden.

4. Department of Pediatrics, Örebro University Hospital, Örebro, Sweden

5. Department of Medicine, Columbia University College of Physicians and Surgeons, New York, New York, USA

6. Faculty of Medicine and Health, School of Medical Sciences, Örebro University, Örebro, Sweden

**Content** 2

Supplementary Methods 3-4

Supplementary Table 1 5

Supplementary Table 2 6

Supplementary Table 3 7

Supplementary Table 4 8

Supplementary Table 5 9-10

Supplementary Table 6 11

Supplementary Table 7 12

Supplementary Table 8 13

Supplementary Figure 1 14

# Supplementary Methods

## The Swedish Twin registry

The STR, established in the late 1960s, includes information on more than 80,000 twin pairs with known zygosity born in Sweden from 1886 onwards.^1^ Zygosity is determined by DNA analysis, opposite sex or a validated algorithm on intra-pair similarity.^2^ The intra-pair similarity algorithm results in a correct classification of zygosity for > 95% of twin pairs compared with DNA analysis.^2^

## Heritability estimations

The theoretical foundation for heritability estimations in twin studies is rooted in the assumption that all individuals within a population are expected to display heterogeneity in their risk of developing a disease. This underlying disease risk, termed disease liability, represents the sum of all genetic and environmental risk factors for the disease. The disease becomes present when disease liability surpasses a (theoretical) threshold level. Heritability is the proportion of the variation in disease liability that can be attributed to variance in genetic factors.

Within the framework of biometrical genetic modelling, the aetiology of a phenotype is attributed to additive genetic effects (A), shared environmental components (C), dominant genetic effects (D) and non-shared environmental effects (E). We used the ACE model to estimate heritability, which equals the proportion of variance captured by A.

It was assumed that "A" sources of variance were shared fully between MZ twins, and to correlate 0.5 between DZ twins, “D” was assumed to correlated 1.0 between MZ twins and 0.25 between DZ twins, "C" factors were supposed to correlate 1.0 between MZ and DZ twins. Conversely, variance source "E" was posited as uncorrelated between individual pairs. Under these assumptions, structural equation modelling was used to partition the variation in the liability of a disease into the A, C and E variables. We also fitted the CE, ADE and AE models using the same methodology. Since the IBD subtypes were treated as mutually exclusive, no bivariate model estimating their etiological overlap was fitted. a, a non-parametric bootstrapping procedure with 1,000 repetitions, resampling twin pairs as clusters, was used to estimate the p-value for the difference in heritability between Crohn's disease and ulcerative colitis, while accounting for potential interdependencies between the IBD subtypes and across twins in pairs.

## Concordance rates

Proband concordance rates is an estimate of the risk of disease in the co-twin of an affected twin (the proband).^3^ It was computed separately for Crohn's disease and ulcerative colitis using a generalised estimating equation with a log-link function. This analysis used a cluster-robust sandwich estimator to compute standard errors while adjusting for dependencies within twin pairs.

## Supplementary references

1. Zagai U, Lichtenstein P, Pedersen NL, et al. The Swedish Twin Registry: Content and Management as a Research Infrastructure. *Twin Res Hum Genet Off J Int Soc Twin Stud* 2019;22:672–680.

2. Lichtenstein P, De Faire U, Floderus B, et al. The Swedish Twin Registry: a unique resource for clinical, epidemiological and genetic studies. *J Intern Med* 2002;252:184–205.

3. McGue M. When assessing twin concordance, use the probandwise not the pairwise rate. *Schizophr Bull* 1992;18:171–176.

**Supplementary table 1.** Previous registry based twins studies of Crohn’s diseae and ulcerative colitis with reports on proband concordance rate

|  |
| --- |

| Study | Study period | Country | IBD twin pairs, n | Concordance rates, CD | Concordance rates, UC | Estimates of heritability, CD | Estimates of heritability, UC |
| --- | --- | --- | --- | --- | --- | --- | --- |
| Tysk, 1988 | 1964-1987 | Sweden | 80 | Monozygotic: 0.58  Dizygotic: 0.04 | Monozygotic: 0.06  Dizygotic: 0.0 | 1.00 | Not computed |
| Orholm, 2000 | 1953-1994 | Denmark | 103 | Monozygotic: 0.58  Dizygotic: 0.0 | Monozygotic: 0.18  Dizygotic: 0.05 | Not reported | Not reported |
| Halfvarson, 2003 | 1964-2001 | Sweden | 80 | Monozygotic: 0.63  Dizygotic: 0.04 | Monozygotic: 0.19  Dizygotic: 0.0 | Not reported | Not reported |
| Jess, 2005 | 1964-2001 | Denmark | 103 | Monozygotic: 0.64  Dizygotic: 0.04 | Monozygotic: 0.18  Dizygotic: 0.05 | Not reported | Not reported |
| Halfvarson, 2011 | 1964-2000 | Sweden | 173 | Monozygotic: 0.38  Dizygotic: 0.02 | Monozygotic: 0.15  Dizygotic: 0.08 | 0.89 | 0.23 |

Abbreviations: IBD, Inflammatory bowel disease; CD, Crohn’s disease; UC, ulcerative colitis

**Supplementary Table 2.** International Classification of Diseases (ICD) codes used to identify patients with inflammatory bowel disease

| ICD-10 | ICD-9 | ICD-8 | ICD-7 |
| --- | --- | --- | --- |
| K51 | 556 | 563.10 | 572.21 |
| K50 | 555 | 563.00 | 572.09 |
| K52.3 |  | 563.99 | 572.20 |
|  |  | 569.02 | 572.00 |
|  |  |  | 578.03 |

**Supplementary Table 3.** International Classification of Diseases (ICD) codes specific for Crohn’s disease

| ICD-10 | ICD-9 | ICD-8 | ICD-7 |
| --- | --- | --- | --- |
| K50 | 555 | 563.10 | 572.09 |
|  |  | 563.00 | 572.00 |

**Supplementary Table 4.** International Classification of Diseases (ICD) codes specific for ulcerative colitis

| ICD-10 | ICD-9 | ICD-8 | ICD-7 |
| --- | --- | --- | --- |
| K51 | 556 | 563.99 | 572.21 |
|  |  | 569.02 | 572.20 |
|  |  |  | 578.03 |

**Supplementary Table 5.** Surgical procedure codes and International Classification of Diseases (ICD) codes indicating Crohn’s disease

| 5/6^th^ edition of KVÅ | 7^th^ edition of KVÅ97 | ICD-10 |
| --- | --- | --- |
| 616 | JHD20 | K603 |
| 629 | JAJ00 | K604 |
| 4630 | JDH32 | K605 |
| 4631 | JFA38 | K610 |
| 4631 | JFA58 | K611 |
| 4640 | JFA60 | K612 |
| 4641 | JFA76 | K613 |
| 4642 | JFA86 | K614 |
| 4643 | JFB00 | K624 |
| 4644 | JFB01 |  |
| 4648 | JFB20 |  |
| 4649 | JFB21 |  |
| 4713 | JFB30 |  |
| 4860 | JFB31 |  |
| 4922 | JFB33 |  |
| 4923 | JFB34 |  |
| 4 970 | JFB40 |  |
| 4999 | JFB41 |  |
|  | JFB43 |  |
|  | JFB44 |  |
|  | JFB46 |  |
|  | JFB47 |  |
|  | JFB50 |  |
|  | JFB51 |  |
|  | JFB60 |  |
|  | JFB61 |  |
|  | JFB63 |  |
|  | JFB64 |  |
|  | JFB96 |  |
|  | JFB97 |  |
|  | JFC00 |  |
|  | JFC01 |  |
|  | JGA58 |  |
|  | JGB10 |  |
|  | JGB11 |  |
|  | JHA00 |  |
|  | JHD21 to JDH 33 |  |
|  | JHD50 to JHD 63 |  |
|  | JHW96 |  |
|  | KCH30 to KCH 33 |  |
|  | LEE30 |  |
|  | XJH00 |  |
|  | JHD20 |  |
|  | JHD30 |  |
|  | JHD33 |  |
|  | JHD50 |  |
|  | JHD60 |  |
|  | JHD63 |  |
|  | JHA00 |  |
|  | JHA20 |  |
|  | JHW96 |  |

Abbreviations: KKÅ: klassifikation av kirurgiska åtgärder (Classification of Surgical Procedures)

**Supplementary Table 6.** Prevalence estimates for Crohn’s disease and ulcerative colitis stratified for sex and zygosity in the Swedish nationwide twin cohort

|  | Total number of twins, n | Twins diagnosed with CD, n | Prevalence of CD, (%) | Twins diagnosed with UC, n | Prevalence of UC, (%) |
| --- | --- | --- | --- | --- | --- |
|  |  |  |  |  |  |
| Monozygotic | 36 090 | 100 | 0.28 | 178 | 0.49 |
| Males | 16 134 | 55 | 0.34 | 91 | 0.56 |
| Females | 19 956 | 45 | 0.23 | 87 | 0.44 |
| Dizygotic | 74 990 | 274 | 0.37 | 446 | 0.59 |
| Males | 36 042 | 137 | 0.38 | 223 | 0.62 |
| Females | 38 948 | 137 | 0.35 | 223 | 0.57 |

Abbreviations: CD, Crohn’s disease; UC, ulcerative colitis

**Supplementary Table 7.** ACE-, CE- ADE- and AE- models for Crohn’s disease

| Model | A + D = Broad-sense heritability (95% CI) | A= Additive genetic effects,  (95% CI) | C = Shared environmental effects,  (95% CI) | E = Unique environmental effects,  (95% CI) | D = Dominant genetic effects,  (95% CI) |
| --- | --- | --- | --- | --- | --- |
| ACE | 0.82  (0.71 – 0.92) | 0.82  (0.71 – 0.92) | 0.00  (0.00 – 0.00) | 0.18  (0.08 – 0.29) |  |
| ACE adjusted† | 0.78  (0.68 – 0.87) | 0.78  (0.68 – 0.87) | 0.00  (0.00 – 0.00) | 0.22  (0.13 – 0.32) |  |
| CE | NA |  | 0.67  (0.55 – 0.79) | 0.33  (0.21 – 0.45) |  |
| CE adjusted† | NA |  | 0.52  (0.42 – 0.61) | 0.48  (0.39 – 0.58) |  |
| ADE* | 0.82  (0.72 – 0.93) | 0.0 |  | 0.18  (0.07 – 0.28) | 0.82  (0.72 – 0.93) |
| ADE* adjusted† | 0.80  (0.72 – 0.89) | 0.0 |  | 0.20  (0.11 – 0.28) | 0.80  (0.72 – 0.89) |
| AE | 0.82  (0.71 – 0.92) | 0.82  (0.71 – 0.92) |  | 0.18  (0.08 – 0.29) |  |
| AE adjusted† | 0.78 (0.68 – 0.87) | 0.78 (0.68 – 0.87) |  | 22 (0.13 – 0.32) |  |

† Adjusted for sex and birth year.

*The ADE model for Crohn's disease attributed the entire heritability estimate to D (dominance genetics), whereas A (additive genetics) was estimated to be 0. These estimates lack biological plausibility and likely caused by the fact that only two DZ pairs were concordant for Crohn's disease.

Note: Confidence bounds of the parameter space were truncated at the boundary (e.g., variance explained was not less than 0).

Abbreviations: CI, confidence interval

**Supplementary Table 8.** ACE-, CE- ADE- and AE- models for ulcerative colitis

| Model | A+ D = Broad-sense heritability (95% CI) | A= Additive genetic effects,  (95% CI) | C = Shared environmental effects,  (95% CI) | E = Unique environmental effects,  (95% CI) | D = Dominant genetic effects,  (95% CI) |
| --- | --- | --- | --- | --- | --- |
| ACE | 0.62 (48 – 77) | 0.62 (48 – 77) | 0.00  (0.00 – 0.00) | 0.38  (0.23 – 0.52) |  |
| ACE adjusted† | 0.57  (0.46 – 0.69) | 0.57  (0.46 – 0.69) | 0.00  (0.00 – 0.00) | 0.43  (0.31 – 0.54) |  |
| CE | NA |  | 0.48  (0.35 – 0.61) | 0.52  (0.39 – 0.65) |  |
| CE adjusted† | NA |  | 0.38  (0.29 – 0.47) | 0.62  (0.53 – 0.71) |  |
| ADE | 0.63  (0.49 – 0.78) | 0.43  (0.00 – 1.00) |  | 0.37  (0.22 – 0.51) | 0.20  (0.00 – 1.0) |
| ADE adjusted† | 0.61  (0.49 – 0.72) | 0.23  (0.00 – 0.77) |  | 0.39  (0.28 – 0.51) | 0.38  (0.00 – 0.95) |
| AE | 0.62  (0.48 – 0.77) | 0.62  (0.48 – 0.77) |  | 0.38  (0.23 – 0.52) |  |
| AE adjusted† | 0.57  (0.46 – 0.69) | 0.57  (0.46 – 0.69) |  | 0.43  (0.31 – 0.54) |  |

† Adjusted for sex and birth year.

Note: Confidence bounds of the parameter space were truncated at the boundary (e.g., variance explained was not less than 0).

Abbreviations: CI, confidence interval

**
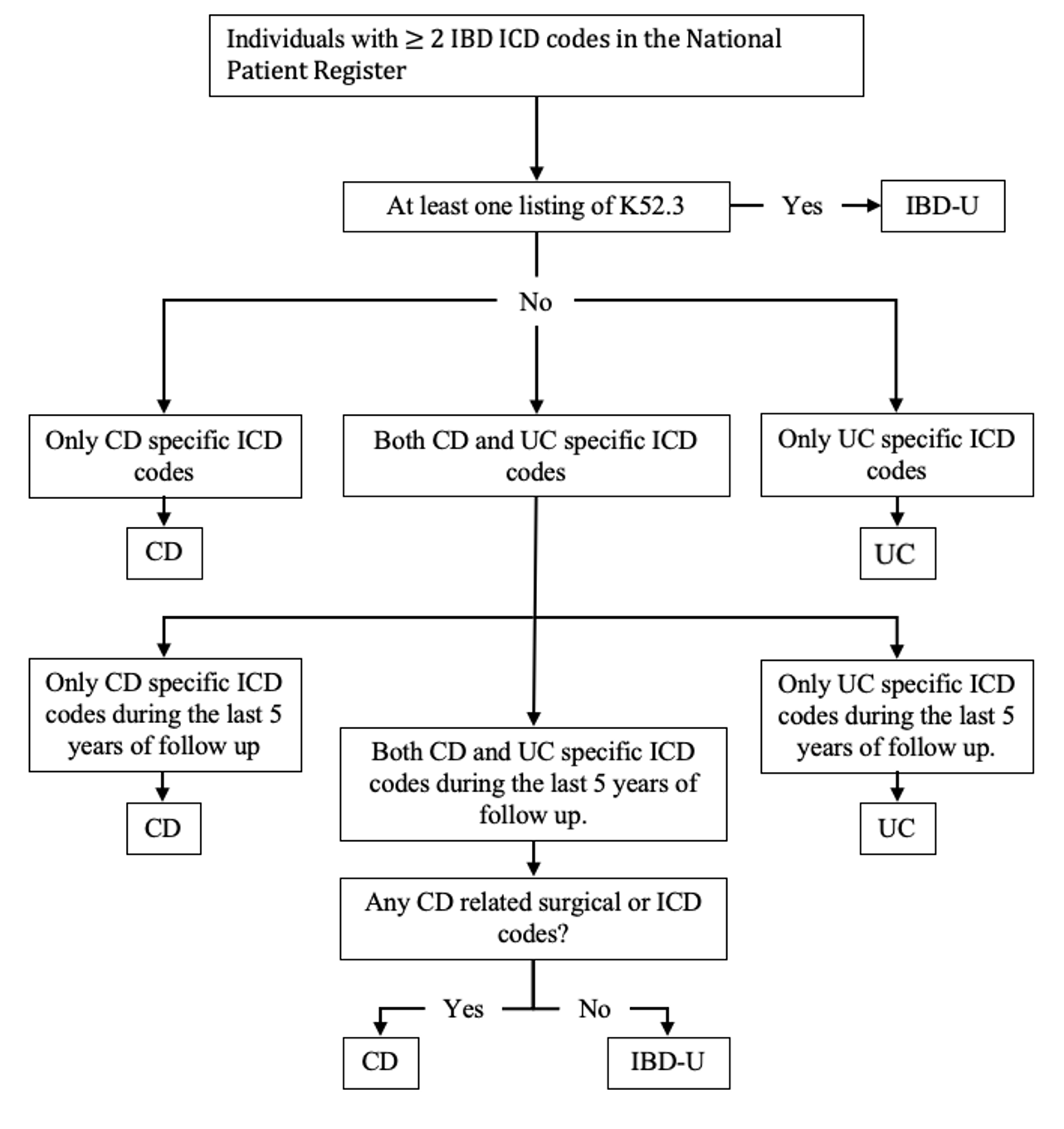
**

**Supplementary Figure 1.** Flowchart showing how International Classification of Diseases (ICD) codes and surgery/procedural codes were used to identify patients with inflammatory bowel disease (IBD) and define IBD subtypes. IBD ICD codes, Crohn’s disease (CD)-specific ICD codes, ulcerative colitis (UC) specific ICD codes and CD-related codes are found in Supplementary Tables 2-5. In this study, we did not stratify the analysis for IBD-unclassified (IBD-U), as a specific ICD code for IBD-U (K52.3) was not used in Sweden before 2007. The last 5 years of follow-up were defined as the 5 years preceding the date of the last registered IBD ICD code in the National Patient Register.
